# Epidemiology and spillover risk of SFTSV in animals across East Asia

**DOI:** 10.71150/jm.2608006

**Published:** 2026-10-31

**Authors:** Yun-Seon Shin, Jeong Yoon Lee, Jun-Gu Kang

**Affiliations:** 1 Korea Zoonosis Research Institute, Jeonbuk National University, Iksan 54596, Republic of Korea; 2 Department of Veterinary and Animal Science, College of Veterinary Medicine, Jeonbuk National University, Iksan 54596, Republic of Korea

**Keywords:** SFTS, animal hosts, spillover risk, tick-borne zoonosis, East Asia

## Abstract

Severe fever with thrombocytopenia syndrome (SFTS) is an emerging tick‑borne zoonosis in East Asia, yet the contribution of animals to virus maintenance and spillover remains incompletely characterized. This review examines current evidence of SFTS virus (SFTSV) infection across domestic livestock, companion animals, wildlife, and captive species, focusing on host range, geographic distribution, diagnostics, and reports of viral RNA and virus isolation. Available data indicate that SFTSV exposure and infection occur across a broad range of animal hosts, supporting a multi-host ecological framework rather than a simple single-reservoir interpretation. Domestic ruminants frequently exhibit high seroprevalence and may function as sentinels of enzootic activity, while occasional virus isolation from cattle demonstrates that viable virus can be recovered from naturally infected livestock, although this does not establish reservoir or amplification competence. Companion animals are considered a potential interface for interspecies transmission, as some studies have documented severe clinical disease, high viral loads, and cat-associated human transmission events supported by varying levels of epidemiological and genomic evidence. Studies in wildlife further document exposure and natural infection across diverse taxa, although their specific roles in viral maintenance remain unresolved. Interpretation of these findings depends heavily on the diagnostic modality: serology primarily indicates prior exposure, RNA detection supports current or recent infection, and virus isolation demonstrates that viable virus was present in the sampled material. Integrating animal infection data within an ecological framework reveals major knowledge gaps related to reservoir competence, infectious shedding, phylogenetic connections among animal, tick, and human cases, and implications for prevention. The review underscores the need for One Health surveillance that incorporates clinical data from companion animals and applies evidence‑based interpretation to improve recognition of animal–human interfaces associated with potential spillover risk.

## Introduction

Severe fever with thrombocytopenia syndrome (SFTS) is an emerging tick-borne infectious disease caused by severe fever with thrombocytopenia syndrome virus (SFTSV), currently classified as *Bandavirus dabieense* in the genus *Bandavirus*, family *Phenuiviridae*, and class *Bunyaviricetes* (ICTV, 2025). The first case of SFTS was reported in China in 2010 (Yu et al., 2011), followed by subsequent reports in the Republic of Korea (ROK), Japan, and Taiwan (Lee et al., 2024; Peng et al., 2020; Takahashi et al., 2014). *Haemaphysalis longicornis* is considered a principal vector of SFTSV and is widely distributed in East Asia, where SFTS is endemic, particularly in China, the ROK, and Japan (Casel et al., 2021; Kim and Park, 2023; Miao et al., 2020). The major clinical manifestations of SFTS include high fever, thrombocytopenia, leukopenia, and gastrointestinal and neurological symptoms (Casel et al., 2021; Seo et al., 2021).

SFTSV is a negative-sense single-stranded RNA (-ssRNA) virus with a tripartite genome consisting of the L (large), M (medium), and S (small) segments (Casel et al., 2021). The L segment encodes the RNA-dependent RNA polymerase (RdRp), which is essential for viral replication. The M segment encodes a glycoprotein precursor that is cleaved by host proteases into two envelope glycoproteins, Gn and Gc. Gn plays a critical role in receptor recognition and viral attachment (Ni et al., 2015), whereas Gc, a class II fusion protein, mediates membrane fusion and subsequent internalization (Halldorsson et al., 2016). These glycoproteins are key determinants of viral entry and major immunogenic targets for vaccine development. The S segment uses ambisense coding, encoding the nucleocapsid protein (NP) and the nonstructural protein (NSs). NP encapsidates viral RNA to form the ribonucleoprotein complex, whereas NSs suppresses host interferon signaling and functions as a major virulence factor (Casel et al., 2021; Hong et al., 2019; Khalil et al., 2021; Kitagawa et al., 2018; Zhou et al., 2013).

The case fatality rate (CFR) of SFTS varies among countries and has been reported as approximately 5.3% in China, 18.7% in the ROK, and 23% in Japan (Kim et al., 2023; Matsuu et al., 2019; Zhan et al., 2017). This regional variation has been discussed in relation to geographic and ecological factors, as well as genotype distribution (Casel et al., 2021). Currently, no effective antiviral agents or licensed vaccines are available for SFTSV infection. Although SFTSV is primarily transmitted by tick bites, human-to-human transmission via direct contact with blood, body fluids, or secretions of infected individuals has been documented. Animal-associated human infections, particularly those involving close contact with infected cats, have also been reported (Casel et al., 2021; Wu et al., 2022; Yamanaka et al., 2020). Accordingly, SFTS is regarded as an important zoonotic disease that requires close surveillance, not only because of vector-borne transmission but also because of potential infection through animal contact.

Although numerous epidemiological and clinical studies have focused on human infection, systematic investigations of SFTSV infection in animals and the ecological roles of different host species remain limited (Casel et al., 2021). Therefore, this review aims to summarize animal species with reported evidence of SFTSV exposure or infection, distinguish among serological, molecular, and virus-isolation evidence, and evaluate their implications without equating detection with reservoir or amplification competence.

## Literature Search Strategy

Relevant studies were identified through searches of PubMed and Google Scholar using the terms “SFTS,” “SFTSV,” “animal,” and “virus isolation,” as well as combinations of these terms with specific host categories, including livestock, companion animals, wildlife, rodents, cats, and dogs. Studies published from database inception through January 2026 were included if they reported serological evidence, viral RNA detection, or virus isolation from naturally infected animals in East Asia and provided sufficient host-specific information, including host species, specimen type, diagnostic method, and numbers tested and positive, for data extraction.

Studies involving experimental infections, focusing exclusively on ticks or humans, or lacking identifiable information on host species, specimen type, diagnostic method, or the numbers of tested and positive animals were excluded from the aggregated analysis. Because study populations, specimen types, diagnostic assays, and sampling designs varied among studies, the combined data were interpreted as descriptive summaries rather than pooled prevalence estimates. In addition, serological and molecular tests were not consistently performed on, or reported for, the same individual animals; therefore, some animals may have been represented in more than one diagnostic category.

## Patterns of SFTSV Infection across Animal Hosts

The animal exposure and infection data summarized in Table 1 indicate that SFTSV has been detected in a wide range of vertebrate hosts across East Asia. Figure 1, based on the data presented in Table 1, provides a schematic overview of SFTSV detection across major animal host groups. It also schematically illustrates potential connections among these hosts through shared tick vectors within the animal–tick–human transmission interface. For the purpose of synthesis, some host categories were grouped at higher taxonomic levels (e.g., “birds” or “rodents”), particularly where individual species were inconsistently reported or where numerous species were represented by limited sample sizes. This grouping was applied to facilitate comparative interpretation across studies. Across more than 40,000 aggregated animal observations reported in studies from multiple endemic countries, serological and molecular evidence have been reported in livestock, companion animals, wildlife, and captive species. Because serological and molecular testing were not consistently performed on the same individuals, this number should not be interpreted as the number of unique animals. The reported magnitude and pattern of detection vary substantially among host groups and study settings (Cheng et al., 2025; Li et al., 2016; Zhao et al., 2022). A key feature of these data is the heterogeneity of diagnostic profiles across species. Some host groups are characterized mainly by serological positivity, whereas others show more frequent RNA detection or occasional virus isolation. These contrasting patterns may reflect differences in exposure and infection dynamics, but they are also influenced by specimen type, diagnostic method, sampling time, study population, and sampling context.

### Livestock

Domestic livestock, particularly cattle, goats, and sheep, are among the most extensively surveyed host groups in endemic regions. Across studies, these species frequently show relatively high seroprevalence, consistent with repeated exposure to SFTSV in tick-endemic agricultural environments (Ding et al., 2014; Kang et al., 2018a; Niu et al., 2013). In the aggregated dataset, cattle constitute one of the largest groups of tested animals, with substantial numbers of seropositive individuals, whereas goats and sheep show similar patterns of antibody detection. By contrast, RNA detection rates in livestock are generally lower than seroprevalence. This pattern, characterized by high antibody positivity and comparatively infrequent RNA detection, has been reported across multiple studies (Chae et al., 2024; Ding et al., 2014; Niu et al., 2013). These findings suggest that although exposure is common, detection of viral RNA at a single time point is less frequent in these species. Virus isolation has also been reported in livestock, including cattle, although such findings remain limited (Chae et al., 2024). Overall, livestock are characterized by frequent serological positivity and comparatively lower molecular positivity in cross-sectional sampling.

### Companion animals

Companion animals, particularly cats and dogs, have been reported to show both serological and molecular evidence of SFTSV infection in endemic regions. In the available literature, RNA detection has been reported more frequently in companion animals than in livestock and some wildlife host groups, particularly among clinically affected individuals (Kang et al., 2019, 2020; Kuan et al., 2023b; Matsuu et al., 2019). In cats, RNA-positive cases have been repeatedly reported in both clinical and field studies, and relatively high viral loads have been observed in naturally infected individuals (Kang et al., 2020; Kuan et al., 2023b; Matsuu et al., 2019). However, this apparent difference should be interpreted cautiously because many companion-animal studies preferentially sampled clinically affected animals, which may have increased the likelihood of detecting viral RNA. In dogs, evidence of exposure, including antibodies and occasional detection of RNA, has also been reported (Ishijima et al., 2023; Kang et al., 2019). However, compared with cats, reports of molecular positivity and clinical severity vary among studies. Overall, companion animals show evidence of both prior exposure and natural infection, but the reported frequency of RNA detection should be interpreted in light of clinical sampling bias and differences in study design.

### Wildlife

Wildlife represents the most taxonomically diverse host category in Table 1 and includes rodents, hedgehogs, wild boars, deer, and various carnivores (Hayasaka et al., 2016; Kaneko et al., 2023; Ni et al., 2015; Yu et al., 2019; Zhao et al., 2022). Evidence of SFTSV exposure and natural infection in wildlife includes serological and molecular findings, although patterns vary substantially among species. Rodents are among the most frequently studied wildlife hosts and have repeatedly been reported as RNA-positive across multiple geographic regions (Gu et al., 2022; Ni et al., 2015). In some studies, virus isolation has also been achieved from rodent species, confirming that a viable virus was present in some naturally infected animals without establishing a reservoir or maintenance competence. Hedgehogs have also been reported to be both seropositive and RNA-positive, and some studies have described virus isolation (Sun et al., 2017; Zhao et al., 2022). Although the number of studies is less than that for rodents, positive findings have been reported across multiple datasets. By contrast, larger mammals such as wild boars and deer are more often characterized by high seroprevalence with lower rates of RNA detection (Hayasaka et al., 2016; Yu et al., 2019). This pattern is similar to that observed in livestock. Other wildlife species, including carnivores, have been reported as seropositive or RNA-positive, but the available data remain limited (Kaneko et al., 2023).

### Summary of host-associated patterns

Taken together, the data summarized in Table 1 show descriptive differences in reported detection profiles across host groups. Livestock and large wild mammals are typically characterized by high seroprevalence with relatively low reported RNA detection, whereas molecular evidence of infection has been reported more frequently in companion animals and some small wildlife species in the available literature. However, these differences should not be interpreted as directly comparable host-specific detection rates, as study designs and sampling contexts varied substantially. Virus isolation has been reported in a subset of hosts, including rodents, hedgehogs, companion animals, and cattle (Chae et al., 2024; Matsuu et al., 2019; Ni et al., 2015; Zhao et al., 2022). These patterns indicate that SFTSV exposure and natural infection have been documented across multiple host groups, with variation in detection type and frequency depending on species, study design, and sampling context (Cheng et al., 2025; Li et al., 2016; Zhao et al., 2022).

## Interpretation of Detection Methods

Interpretation of animal SFTSV data requires careful attention to the type of diagnostic evidence reported because serology, RNA detection, and virus isolation do not represent equivalent biological evidence of host infection or transmission potential. Although these methods are often presented together in animal surveillance studies, each captures a different dimension of host–virus interaction. Serological positivity indicates prior exposure, molecular detection supports current or recent infection by demonstrating the presence of viral nucleic acid at the time of sampling, and virus isolation provides direct evidence that a viable, replication-competent virus was present in the sampled material (Casel et al., 2021; Jiao et al., 2012; Kim and Park, 2023). Accordingly, interpretation of host-associated SFTSV data depends not only on whether a species is reported as positive, but also on how that positivity was established.

This distinction is particularly important in the context of the broad host range summarized in Table 1. The literature includes species with high seroprevalence but limited RNA detection, species with repeated molecular detection but less extensive serological support, and a smaller subset from which viable virus has been isolated following natural infection. These contrasting diagnostic profiles have different interpretive implications. Diagnostic modality should therefore be treated as a central component of interpretation rather than as a minor technical detail. However, none of these diagnostic findings alone establishes reservoir, amplification, or onward-transmission competence.

### Serological evidence as an indicator of cumulative exposure

Serological assays have been the most widely used tools in animal SFTSV research, particularly in livestock and wildlife studies, because they are practical for large-scale field surveillance and useful for estimating cumulative exposure within host populations (Ding et al., 2014; Hayasaka et al., 2016; Kang et al., 2018a; Niu et al., 2013; Yu et al., 2019). Across endemic regions, Enzyme-linked immunosorbent assay (ELISA), Immunofluorescence assay (IFA), Virus neutralization test (VNT), Gaussia luciferase immunoprecipitation system (GLIPS), Focus reduction neutralization test (FRNT), Immunoperoxidase test (IPT), Single-Round Infectious Particles neutralization assay (SRIP neutralization assay), and related platforms have revealed substantial seropositivity in cattle, goats, sheep, deer, and wild boars, consistent with repeated exposure to SFTSV in tick-endemic environments (Ding et al., 2014; Hayasaka et al., 2016; Niu et al., 2013; Yu et al., 2019). The principal strength of serology is its ability to capture cumulative exposure within host populations. High seroprevalence across multiple studies is compatible with repeated exposure within local transmission environments, although the frequency and route of exposure cannot be determined from serology alone. This pattern is especially evident in domestic ruminants and large wild mammals, in which antibody prevalence is often substantial even when RNA detection is less frequent (Ding et al., 2014; Niu et al., 2013). In this sense, serological surveys are useful for identifying areas of enzootic circulation and recognizing host populations with substantial cumulative exposure. At the same time, seropositivity has clear interpretive limits. Antibody detection cannot distinguish recent from remote infection, cannot determine whether the host was viremic at the time of sampling, and does not establish whether infectious virus remains present. In cross-sectional studies, antibody-positive animals may have cleared infection long before sampling occurred (Ding et al., 2014; Niu et al., 2013). For that reason, serological evidence is best interpreted as evidence of prior exposure rather than proof of current infectiousness. This limitation becomes especially important when discussing reservoir hosts. High seroprevalence indicates repeated exposure within areas of viral circulation but does not define the functional role of a species or demonstrate reservoir competence. Demonstrating reservoir or amplification competence would require evidence that the host sustains viral replication at levels and for a duration sufficient to support onward transmission. Serological evidence alone cannot establish that point (Cheng et al., 2025; Li et al., 2016; Zhao et al., 2022). Accordingly, species supported only by antibody data are more appropriately described as exposed hosts than as confirmed reservoirs.

### Molecular detection as evidence of current or recent infection

Compared with serology, detection of SFTSV RNA by reverse transcription polymerase chain reaction (RT-PCR), qRT-PCR, nested RT-PCR, or related assays demonstrates the presence of viral RNA in the tested specimen at the time of sampling and therefore supports current or recent infection (Chae et al., 2024; Gu et al., 2022; Kang et al., 2019, 2020; Matsuu et al., 2019). In the studies summarized in Table 1, RNA detection has been particularly important for documenting current or recent infection in host groups including cats, dogs, rodents, and cattle (Chae et al., 2024; Gu et al., 2022; Kang et al., 2019, 2020; Matsuu et al., 2019). However, RNA positivity is not equivalent to infectiousness. Detection of viral genetic material does not necessarily mean that an intact, viable virus is still present. Viral RNA may remain detectable after the infectious virus is no longer recoverable, particularly when sampling occurs late in infection or under conditions in which nucleic acid persistence is longer than the persistence of viable virus (Casel et al., 2021; Jiao et al., 2012; Kim and Park, 2023). Thus, molecular positivity provides stronger support than serology for current or recent infection, but does not provide the same evidence of viable virus as successful virus isolation. Interpretation of RNA detection is also strongly influenced by specimen type. As shown in Table 1, animal studies have used serum, plasma, whole blood, spleen, visceral tissues, and, in some companion animal studies, oral, ocular, or rectal specimens. These differences matter because the probability of detection depends on tissue distribution, viral burden, and timing of collection. Whole blood may capture viremia not detected in serum, whereas tissue-based sampling may yield higher positivity in necropsy-based wildlife studies. In companion animals, detection in non-blood specimens may indicate the presence of viral RNA in specimens that are potentially associated with shedding, although it does not, by itself, demonstrate infectious shedding (Ando et al., 2021; Kang et al., 2020; Matsuu et al., 2019). Reported RNA positivity should therefore not be interpreted as directly comparable across studies without accounting for specimen type, collection timing, and sampling context.

Timing of sampling is a second major determinant. Because many animal studies are cross-sectional, they capture only a narrow window of the infection course. If viremia is brief, naturally infected animals may be antibody-positive but PCR-negative at the time of sampling. This helps explain the recurrent pattern observed in livestock and some wildlife hosts: relatively high seroprevalence accompanied by lower RNA detection (Chae et al., 2024; Ding et al., 2014; Kang et al., 2018a; Niu et al., 2013). Similar profiles have been reported in wild boars and deer (Hayasaka et al., 2016; Yu et al., 2019). Such discrepancies do not necessarily indicate limited epidemiological relevance; they may instead reflect the transient window of RNA detectability. This issue is particularly useful for interpreting host-group differences. In cattle, goats, and sheep, high seroprevalence with comparatively infrequent RNA positivity may be consistent with repeated exposure and a limited period during which viral RNA is detectable (Chae et al., 2024; Ding et al., 2014; Kang et al., 2018a; Niu et al., 2013). By contrast, in companion animals such as cats, RNA detection is more often captured in clinically affected individuals, although this pattern may partly reflect the preferential sampling of clinically affected animals (Ando et al., 2021; Kang et al., 2020; Matsuu et al., 2019).

### Virus isolation as evidence of a viable virus

Among the available diagnostic approaches, virus isolation provides the strongest biological evidence that viable, replication-competent SFTSV was present in the sampled material and could be recovered under laboratory conditions, beyond evidence of prior exposure or viral nucleic acid alone (Casel et al., 2021; Kim and Park, 2023; Yishuang et al., 2021). However, virus isolation alone does not establish that the sampled animal transmitted the virus under natural conditions or that the host species possesses reservoir or amplification competence. This distinction is central when considering the potential epidemiological role of an infected animal.

Isolation from *Apodemus agrarius* and hedgehogs demonstrates that viable SFTSV was present in some naturally infected animals. These findings identify these species as candidates for further ecological investigation but do not establish competence in maintenance or amplification. Demonstrating such roles would require evidence of sustained viremia, efficient host-to-tick transmission, and onward transmission under natural conditions.

Isolation from domestic cats is particularly notable because it is supported by a broader evidentiary pattern that includes repeated RNA detection, severe clinical disease, and cat-associated human transmission events. In one nosocomial outbreak, viable SFTSV was isolated from an infected cat and two veterinary personnel, and the complete S, M, and L genome segments showed 100% nucleotide identity, providing strong evidence of direct cat-to-human transmission (Yamanaka et al., 2020). However, even in this strongly supported event, the specific route of exposure—whether through blood, other body fluids, mucosal contact, droplets, or aerosols—was not determined. In this context, isolation is not merely a laboratory finding but part of a broader evidentiary pattern indicating that clinically affected cats may represent a relevant interface for potential human exposure during close contact with blood or body fluids. Isolation from cattle is also significant because it extends the available evidence beyond serological exposure alone. Recovery of viable virus from naturally infected cattle demonstrates that viable SFTSV can occasionally be recovered from livestock under field conditions (Chae et al., 2024). However, this finding remains insufficient to classify cattle or other livestock as reservoir or amplifying hosts.

At the same time, negative isolation findings must be interpreted cautiously. Virus recovery is technically demanding and depends on viral load, specimen type and quality, timing of collection, preservation conditions, and cell-culture methods. Failure to isolate SFTSV, therefore, does not necessarily demonstrate that a viable virus was absent or that the host lacks epidemiological relevance (Casel et al., 2021; Kim and Park, 2023). For that reason, positive isolation should be treated as strong evidence that a viable virus was present in the sampled material, whereas the absence of isolation should not be taken as proof of epidemiological irrelevance. Overall, successful isolation provides strong evidence that viable virus was present in the sampled material, but its ecological significance must be evaluated together with infection duration, shedding, host-to-tick transmission, contact patterns, and evidence of natural onward transmission.

### Diagnostic heterogeneity and its implications for interpretation

A major challenge in synthesizing the animal SFTSV literature is the marked heterogeneity in study design and diagnostic strategy across host species, countries, and sampling settings. This heterogeneity affects not only reported positivity estimates but also the apparent relative importance of host groups. Some studies rely solely on serology, others combine serology with PCR, and only a minority attempt virus isolation. Assay platforms also vary substantially, including ELISA, IFA, VNT, RT‑PCR, nested RT‑PCR, and qRT‑PCR (Huang et al., 2019; Yishuang et al., 2021). Specimen heterogeneity further complicates interpretation. Some studies use serum only, whereas others analyze blood, plasma, spleen, viscera, or multiple specimen types. These differences are not trivial, as a PCR-negative result from one specimen type may not exclude a positive result from another. Companion animal studies that include non-blood specimens may detect viral RNA in oral, ocular, or rectal samples that serum-based surveys would not assess. However, RNA detection in these specimens does not by itself demonstrate infectious shedding (Ando et al., 2021; Matsuu et al., 2019). As a result, direct comparison of RNA detection rates across species is often difficult unless sampling frameworks are closely aligned. Clinical sampling context is another important source of variation. Many livestock and wildlife studies are based on apparently healthy field-caught or farmed animals, whereas some companion animal studies preferentially include clinically ill animals presented to veterinary settings. This difference can substantially influence the likelihood of RNA detection or virus isolation. Some of the apparent contrast between companion animals and other host groups may therefore reflect differences in sampling context as well as possible biological differences in infection dynamics. Geographic and ecological variation adds a final layer of complexity. Host density, habitat structure, agricultural practices, and local vector activity all influence the probability of exposure and detection (Cheng et al., 2025; Li et al., 2016). Because serological and molecular testing was not consistently performed on or reported for the same individual animals, the aggregated sample sizes in Table 1 may include overlap across diagnostic categories. The totals should therefore be interpreted as aggregated observations rather than counts of unique animals, independent denominators, or pooled sample sizes. Taken together, diagnostic results should be interpreted within their methodological and ecological settings rather than treated as directly interchangeable across studies.

### An evidence-weighted framework for interpretation

Taken together, the available data support an evidence-weighted framework for interpreting animal SFTSV findings. Serology, RNA detection, and virus isolation provide complementary but non-equivalent evidence of exposure, current or recent infection, and the presence of viable virus, respectively; none alone establishes natural transmission, reservoir competence, or amplification competence (Casel et al., 2021; Kim and Park, 2023; Yishuang et al., 2021). This evidence-weighted approach helps avoid two common interpretive errors. The first is overinterpretation of serology, in which antibody-positive species are prematurely described as reservoirs or major transmission hosts despite lacking evidence of active or infectious virus. The second is the underinterpretation of stronger virological evidence, particularly when repeated RNA detection or virus isolation is treated as no more informative than serological exposure. A balanced interpretation is to describe serology-only species as exposed hosts, species with repeated molecular detection as hosts with evidence of current or recent infection, and isolation-positive species as hosts in which viable virus has been documented (Chae et al., 2024; Cheng et al., 2025; Zhao et al., 2022). These categories are complementary and should be interpreted together. The key question is not simply whether a host species has tested positive, but what type of positivity was observed, in which specimen, under what sampling conditions, and with what degree of biological confidence. Only by applying this layered interpretive approach can animal infection data be used to formulate cautious hypotheses regarding maintenance, amplification, and spillover, while recognizing that these hypotheses require additional ecological and transmission evidence across the animal–tick–human interface.

## Transmission Implications

The epidemiological significance of animal SFTSV infection extends beyond vector-borne ecology alone. Although infected ticks remain the principal route of transmission in nature, available evidence indicates that direct exposure to infected blood or body fluids may result in transmission under certain circumstances, particularly at the animal–human interface (Hu et al., 2020; Yamanaka et al., 2020). The public health relevance of an infected animal, therefore, depends not only on its infection status but also on the opportunities for exposure created during handling, clinical care, restraint, necropsy, or contact with blood and other body fluids. Transmission implications should accordingly be interpreted in relation to host infection status, contact intensity, clinical context, and the evidentiary strength supporting non-vector spread (Kida et al., 2019; Kim and Park, 2023; Yamanaka et al., 2020).

### Animal-to-human transmission

Reports of animal-to-human transmission of varying evidentiary strength have primarily involved companion animals, particularly cats. The evidentiary strength of reported events was assessed using epidemiological linkage, exclusion of alternative tick exposures, viral sequence identity, virus isolation, and availability of whole-genome comparisons. Case reports and outbreak investigations indicate that humans may be exposed to SFTSV through close contact with infected cats in veterinary settings, where contact with blood, saliva, respiratory secretions, or other body fluids may occur, although the specific infectious material responsible for transmission has not been identified (Ando et al., 2021; Kida et al., 2019; Yamanaka et al., 2020). These findings are epidemiologically relevant because they demonstrate the potential for zoonotic exposure in real clinical settings. The public health implications of feline infection have been further discussed in light of reports describing severe systemic disease and relatively high viral loads in naturally infected cats (Ando et al., 2021; Matsuu et al., 2019; Osako et al., 2024). Under such conditions, bites, scratches, mucosal exposure, and direct contact with blood or body fluids that may contain the virus have been proposed as possible routes; however, strongly supported transmission events have also occurred in the absence of recognized bites or scratches. Accordingly, clinically affected cats may serve as a relevant interface for interspecies exposure, as close contact with humans often occurs during veterinary care (Ando et al., 2021; Kida et al., 2019; Yamanaka et al., 2020). The reported cat-associated events differ in evidentiary strength. One report described a veterinarian who developed SFTS after caring for and performing autopsies on infected cats; an identical 421-nucleotide viral sequence was identified in the patient and two cats, although the specific source cat, transmission route, and whole-genome linkage were not established (Kida et al., 2019). By contrast, a nosocomial outbreak involving an infected cat and two veterinary personnel provided stronger virological evidence: viable virus was isolated from all three individuals, and the complete S, M, and L genome segments showed 100% nucleotide identity (Yamanaka et al., 2020). This outbreak therefore provides stronger evidence of direct cat-to-human transmission, although the precise route of exposure remains undetermined. Dogs have also been associated with human exposure under certain circumstances, although evidence for direct dog-to-human transmission remains limited and is less extensive than that for cats. Naturally infected dogs have been reported as RNA-positive and clinically affected in endemic regions (Kang et al., 2019; Nam et al., 2020), and a human infection temporally associated with close contact during tick removal has also been described (Chung et al., 2020). However, exposure to the infected tick cannot be clearly distinguished from direct transmission from the dog in this setting. While these findings do not support classifying dogs at the same level as cats in terms of evidence for zoonotic spillover, they suggest that dogs may be associated with human exposure in specific contexts, particularly during tick removal or close handling of clinically affected animals (Chung et al., 2020; Kang et al., 2019; Nam et al., 2020). Taken together, zoonotic transmission risk appears to depend not only on host susceptibility but also on patterns of contact, route of exposure, and environmental context. Companion animals live in settings characterized by close human contact, and in some situations, infections in these hosts may more readily translate into human exposure. These considerations highlight the importance of including companion animals in assessments of interspecies transmission risk, while distinguishing events by their evidentiary strength (Ando et al., 2021; Kida et al., 2019; Yamanaka et al., 2020).

### Animal-to-animal transmission

Compared with animal-to-human transmission, direct evidence for animal-to-animal transmission remains limited. Nevertheless, non-vector animal-to-animal transmission is biologically plausible under certain conditions (Chae et al., 2024; Matsuu et al., 2019; Zhao et al., 2022). Recovery of viable virus from naturally infected animals indicates that replication-competent SFTSV was present in the sampled material, suggesting that close contact among animals could permit non-vector transmission under appropriate conditions (Chae et al., 2024; Matsuu et al., 2019; Zhao et al., 2022). However, virus isolation alone does not demonstrate direct animal-to-animal transmission. This possibility may be particularly relevant in companion animal settings. Infected cats can develop severe disease accompanied by high viral burdens, creating conditions in which contact with blood, saliva, ocular discharge, or other body fluids may plausibly expose other animals in shelters, veterinary hospitals, or multi-animal households (Ando et al., 2021; Matsuu et al., 2019; Osako et al., 2024). The reported cat-to-human events, including the genomically linked outbreak, do not demonstrate direct cat-to-cat transmission, and no equivalent genomic linkage between naturally infected animals has been established in these reports. However, current field evidence remains insufficient to determine how often such direct transmission occurs or whether it contributes meaningfully to sustained transmission outside periods of tick activity. A similar uncertainty applies to wildlife. The recovery of viable virus from wildlife hosts such as rodents or hedgehogs confirms natural infection but does not establish non-vector animal-to-animal transmission (Ni et al., 2015; Zhao et al., 2022). Moreover, most field studies cannot distinguish direct transmission from shared exposure to infected ticks. As a result, animal-to-animal spread remains a plausible but incompletely defined component of SFTSV transmission ecology rather than a firmly established maintenance route.

### Non-vector transmission and occupational implications

Available evidence indicates that tick-borne transmission remains the dominant natural pathway of SFTSV circulation (Hu et al., 2020; Zhuang et al., 2018). Nevertheless, documented person-to-person transmission through exposure to infected blood demonstrates that contact-based transmission is biologically possible and provides a relevant framework for interpreting animal-associated exposure (Wu et al., 2022). The precise route involved in individual animal-associated events often remains uncertain, and the relative contributions of blood, other body fluids, mucosal exposure, droplets, or aerosols have not been fully resolved.

The possibility of non-vector transmission is particularly relevant in clinical and occupational settings. Clinically affected animals with high viral loads may create opportunities for exposure during examination, treatment, restraint, specimen collection, necropsy, or handling of materials potentially contaminated with blood or body fluids. Veterinarians, veterinary nurses, shelter workers, farmers, wildlife handlers, and pet owners may therefore encounter blood or other body fluids that may contain viral RNA and, in some reported cases, viable virus (Ando et al., 2021; Chung et al., 2020; Kida et al., 2019; Yamanaka et al., 2020).

Prevention strategies should therefore address both tick-borne transmission and possible contact-based exposure. In settings involving clinically ill animals, appropriate personal protective equipment, careful handling of blood and body fluids, safe specimen collection, and precautions during necropsy are warranted (Ando et al., 2021; Kida et al., 2019; Yamanaka et al., 2020). These measures are particularly relevant when the infection status of an animal is unknown but clinical and epidemiological features are compatible with SFTSV infection.

Overall, non-vector transmission is less frequently documented than tick-borne transmission, but its true frequency, specific routes, and broader epidemiological contribution remain uncertain. This uncertainty emphasizes the need for improved infection-control awareness and systematic investigation of occupational and veterinary-associated exposures.

## Ecological and One Health Implications

The broad host range and heterogeneous infection profiles summarized in this review are consistent with SFTSV circulation within a multi-host ecological system rather than a simple single-reservoir structure (Cheng et al., 2025; Li et al., 2016; Zhao et al., 2022). Across East Asia, SFTSV exposure and natural infection have been documented in livestock, companion animals, and diverse wildlife species, although the type and strength of evidence differ substantially among host groups. Collectively, these findings suggest a transmission network in which host roles vary with ecological setting, vector activity, host abundance, and opportunities for human contact (Cheng et al., 2025; Hu et al., 2020; Zhuang et al., 2018). This interpretation shifts attention away from the search for a single definitive reservoir and toward interactions among hosts, ticks, and human exposure settings. Current evidence is more consistent with a framework in which different vertebrate hosts occupy different positions within the broader transmission system (Cheng et al., 2025; Li et al., 2016; Zhao et al., 2022). Within this framework, livestock may be particularly useful as indicators of enzootic exposure. Domestic ruminants repeatedly show substantial serological evidence of exposure in endemic agricultural settings (Ding et al., 2014; Niu et al., 2013), making them useful for identifying areas with substantial enzootic exposure. Wildlife may contribute to broader ecological circulation, although the extent and nature of this contribution remain species- and context-dependent (Cheng et al., 2025; Li et al., 2016; Zhao et al., 2022). Companion animals occupy a different position: their clearest documented significance lies at a high-contact interface where animal infection may become directly relevant to human exposure (Ando et al., 2021; Kida et al., 2019; Yamanaka et al., 2020). These distinctions have direct implications for surveillance. If SFTSV circulation is understood within a multi-host framework, surveillance limited to human cases or infected ticks will capture only part of the transmission landscape. Human case surveillance identifies recognized disease, and tick surveillance reflects vector infection pressure, whereas animal surveillance provides complementary information on host exposure, current or recent infection, and potential spillover-relevant interfaces (Cheng et al., 2025; Kuan et al., 2023a; Li et al., 2016). Different animal sectors may therefore serve different surveillance purposes: livestock serology may be useful for mapping enzootic activity, whereas molecular detection or virus isolation in companion animals and selected wildlife hosts may be more informative for identifying host groups or settings in which current infection or viable virus has been documented (Chae et al., 2024; Zhao et al., 2022). Taken together, these patterns reinforce the need for a One Health framework integrating wildlife ecology, veterinary medicine, livestock management, vector biology, and human public health (Casel et al., 2021; Kim and Park, 2023). Spillover risk may emerge from the overlap of these domains rather than from any one domain alone: infected ticks feed on multiple vertebrate hosts, infected animals may, under some circumstances, create opportunities for direct exposure, and local ecological conditions shape how often animals, vectors, and humans come into contact (Hu et al., 2020; Kida et al., 2019; Yamanaka et al., 2020). For this reason, preparedness is likely to be most effective when animal surveillance, tick monitoring, veterinary awareness, and human public health responses are treated as connected rather than separate systems (Casel et al., 2021; Cheng et al., 2025; Kim and Park, 2023). From a practical perspective, this integrated approach is especially relevant in farms, veterinary clinics, animal shelters, and wildlife management settings, where animal infection may function both as an ecological signal and as a potential occupational concern (Ando et al., 2021; Chung et al., 2020; Kida et al., 2019; Yamanaka et al., 2020). In such environments, an infected animal may not only indicate local circulation of SFTSV but, in some circumstances, may also identify a setting in which spillover-relevant exposure could occur.

Overall, SFTSV ecology is best interpreted through a host–vector–human framework in which different animal groups occupy different positions in ecological circulation and spillover-relevant interfaces (Cheng et al., 2025; Li et al., 2016; Zhao et al., 2022). One Health surveillance should therefore focus not only on infected species, but also on the interfaces that connect enzootic circulation with human exposure risk (Casel et al., 2021; Cheng et al., 2025; Kim and Park, 2023).

## Knowledge Gaps

Despite the growing literature on animal SFTSV infection, major uncertainties remain regarding which host species may contribute to viral maintenance, which primarily reflect exposure, and which may be most relevant to spillover into humans (Cheng et al., 2025; Li et al., 2016; Zhao et al., 2022). Current studies have established that a wide range of animals can be seropositive, RNA-positive, and in some cases, yield viable virus upon isolation. However, these findings do not by themselves identify the hosts that matter most epidemiologically. In other words, the field has advanced substantially in documenting host breadth, but it still lacks comparable resolution in defining host function.

One of the most important unresolved issues is reservoir competence. Evidence of exposure or infection does not necessarily indicate that a host can efficiently infect feeding ticks, sustain transmission over time, or consistently contribute to local maintenance cycles (Cheng et al., 2025; Li et al., 2016). This issue is especially relevant for wildlife species that are often discussed as putative reservoirs on the basis of repeated field detection alone. Rodents remain plausible candidates because of their abundance, wide distribution, and repeated molecular detection (Gu et al., 2022; Ni et al., 2015), whereas hedgehogs have attracted particular attention because repeated field detection and virus isolation identify them as candidates for further investigation of their potential amplification roles (Zhao et al., 2022). However, direct evidence of efficient onward transmission remains insufficient, and for most wildlife hosts, current evidence does not clarify whether, or how consistently, such roles operate across ecological settings.

A second major gap concerns duration of viremia, shedding of viable virus, and host infectiousness. Most animal studies are cross-sectional and therefore identify whether infection has occurred, but not how long infected animals remain viremic, have detectable viral RNA, shed viable virus, or create opportunities for transmission (Ando et al., 2021; Chae et al., 2024; Matsuu et al., 2019). This limitation is particularly important at the animal–human interface. The public health relevance of an infected animal depends not only on whether the virus is detectable at a single time point, but also on whether viable virus is present, in what quantity, in which specimens or body fluids, and for how long it remains detectable or recoverable. Without longitudinal data, estimates of host-specific windows of potential infectiousness remain uncertain.

A third unresolved issue is comparative host importance. The available literature suggests that infected hosts are not epidemiologically equivalent, yet direct comparative data remain scarce (Chae et al., 2024; Cheng et al., 2025; Zhao et al., 2022). Some host groups appear to be characterized mainly by repeated exposure, whereas others show stronger evidence supporting current or recent infection or potential spillover relevance. However, these differences are still inferred largely from separate studies that vary in design, specimen type, and sampling context. As a result, it remains difficult to reliably compare host species in terms of their potential contributions to maintenance, amplification, or spillover.

Another major gap concerns phylogenetic linkage across hosts, ticks, and humans. Although several studies have reported molecular detection and sequence analysis in animals (Lee et al., 2017), integrated datasets directly connecting viral sequences across wildlife, livestock, companion animals, ticks, and human cases remain limited. Although whole-genome identity has been demonstrated in a small number of veterinary-associated cat-to-human events, comparable genomic linkage remains unavailable for most animal-associated infections and for most proposed animal-to-animal or host-to-tick transmission pathways. This restricts inference about transmission direction, bridge hosts, and the extent to which infections detected in different sectors belong to the same local transmission cycle (Cheng et al., 2025; Li et al., 2016). More comprehensive cross-sector phylogenetic analyses would substantially improve the interpretation of how enzootic circulation translates into human exposure or disease risk. Methodological heterogeneity further complicates the field. Animal studies vary widely in serological platforms, molecular assays, specimen types, host clinical status, and sampling designs (Huang et al., 2019; Matsuu et al., 2021; Yishuang et al., 2021). These differences weaken comparability across host species, countries, and study settings and may partly explain apparent variation in reported positivity or host importance. Some observed contrasts are likely biological, but others may reflect methodological inconsistency rather than underlying ecological differences. Greater standardization in sampling and diagnostic strategy is therefore essential if future studies are to support stronger comparative inference.

Taken together, these gaps limit the extent to which current animal infection data can be translated into precise surveillance priorities and prediction of spillover risk. Future work should move beyond descriptive host lists and focus more directly on reservoir competence, longitudinal shedding dynamics, comparative host infectiousness, and integrated phylogenetic linkage across the animal–tick–human interface (Cheng et al., 2025; Li et al., 2016; Zhao et al., 2022). Progress in these areas will be necessary to transform the current literature from a catalog of host detections into a mechanistic understanding of how SFTSV persists and spills over in real ecological systems.

## Conclusion

The evidence reviewed here indicates that SFTSV circulates across a wide range of animal hosts in East Asia and is more appropriately understood within a multi-host ecological system rather than a single-reservoir framework (Cheng et al., 2025; Li et al., 2016). Although animal exposure and infection are widely reported, their epidemiological significance varies according to the type of diagnostic evidence. Serological findings primarily reflect prior exposure, molecular detection supports current or recent infection, and virus isolation demonstrates that viable virus was present in the sampled material. However, none of these diagnostic findings alone establishes reservoir, amplification, or onward-transmission competence. Companion animals, particularly cats, occupy a relatively important position because natural infection, clinical disease, virus isolation, and genomically supported cat-associated human transmission have all been documented (Ando et al., 2021; Kida et al., 2019; Matsuu et al., 2019; Yamanaka et al., 2020). In contrast, livestock often reflect enzootic exposure at the landscape level, whereas findings in wildlife document broad exposure and natural infection but do not yet define species-specific maintenance roles (Chae et al., 2024; Cheng et al., 2025; Ni et al., 2015; Zhao et al., 2022).

Taken together, these findings highlight three key points. First, host roles should be interpreted within a multi-host ecological framework rather than assigned to a single presumed reservoir. Second, host importance must be evaluated using an evidence-weighted approach that distinguishes prior exposure from current or recent infection and from the presence of viable virus. Third, spillover risk is shaped not only by host infection status but also by the position of that host within the animal–tick–human interface (Cheng et al., 2025; Kim and Park, 2023; Li et al., 2016). Accordingly, effective preparedness requires a One Health approach that integrates animal surveillance, veterinary recognition, vector monitoring, and human public health responses (Cheng et al., 2025; Kim and Park, 2023; Li et al., 2016). Future research should move beyond descriptive reports of host detection and focus more directly on reservoir competence, infectiousness, shedding dynamics, and cross-sector transmission linkages. Addressing these questions will be essential for identifying the hosts and interfaces that may contribute most meaningfully to viral maintenance and spillover in East Asia (Cheng et al., 2025; Li et al., 2016; Zhao et al., 2022).

## Figures and Tables

**Fig. 1.**
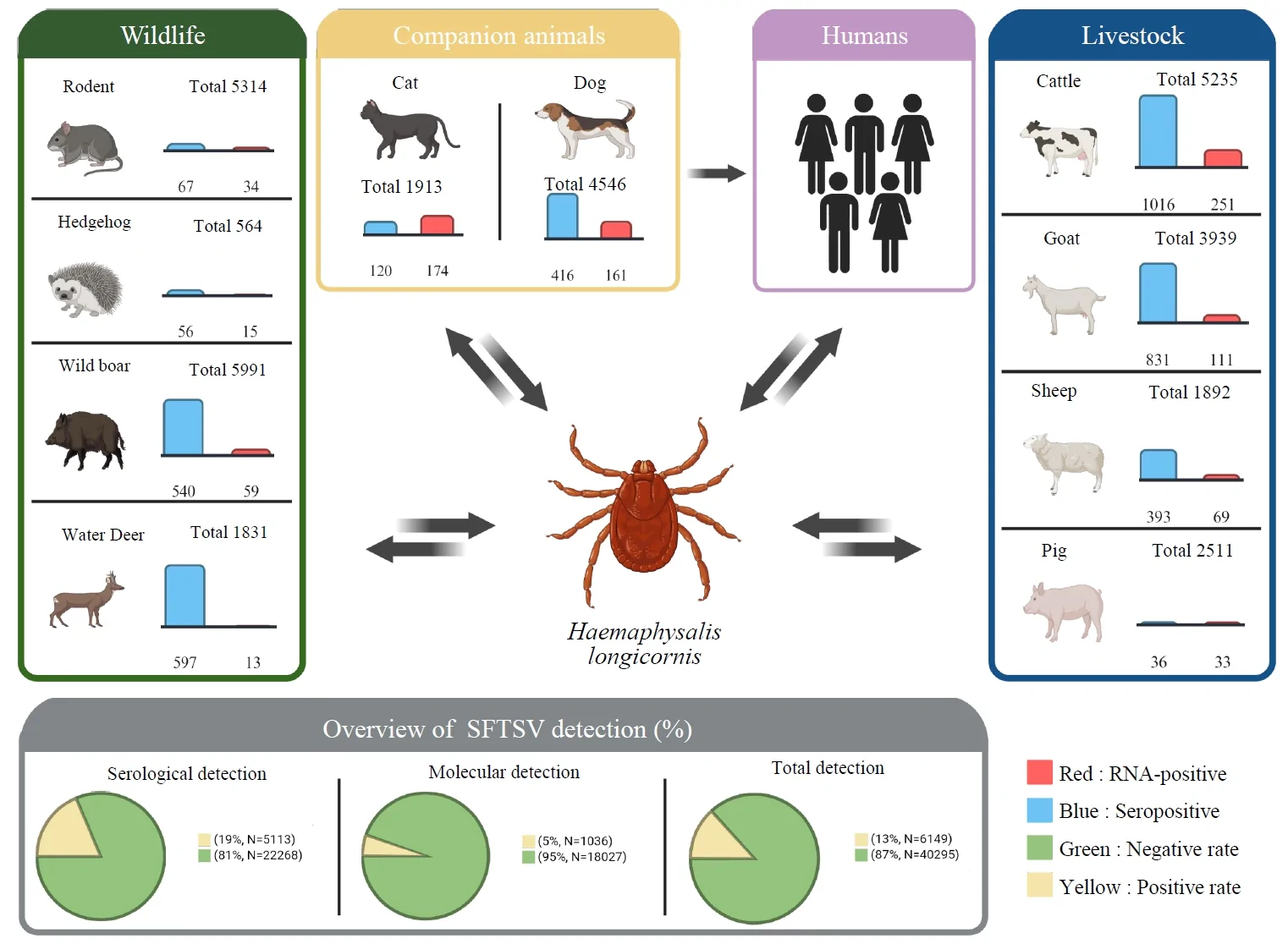
Overview of SFTSV detection across animal hosts and the animal–tick–human interface. This figure summarizes the reported serological and molecular detection of severe fever with thrombocytopenia syndrome virus (SFTSV) across major animal host groups, including wildlife, companion animals, and livestock. Blue bars represent the number of seropositive animals, whereas red bars indicate the number of RNA-positive animals, with total sample sizes provided for each species. The principal tick vector, *Haemaphysalis longicornis*, is centrally positioned to illustrate its role in linking animal hosts and humans within the transmission system. The lower panel presents an aggregated overview of SFTSV detection, showing proportions based on serological detection, molecular detection, and their combined total. Green and yellow sectors denote negative and positive proportions.

**Table 1.** Summary of SFTSV detection in animal hosts across East Asia by country, host species, specimen type, and diagnostic method

Country	Host species	Specimen type	Detection method	Serology (+/tested)		RNA detected (+/tested)		Reference
China	Bird (unspecified)	Serum	ELISA, qRT-PCR	7	155	–	–	Li et al. (2016)
	Cattle	Serum, Blood, Viscera	ELISA, qRT-PCR	828	1501	48	909	Cheng et al. (2025); Huang et al. (2019); Ishijima et al. (2023); Jiao et al. (2012); Li et al. (2014); Niu et al. (2013); Xing et al. (2016)
	Chicken	Serum, Viscera	ELISA, qRT-PCR	344	1398	25	609	Cheng et al. (2025); Huang et al. (2019); Ishijima et al. (2023); Jiao et al. (2012); Li et al. (2014); Niu et al. (2013)
	Dog	Serum, Plasma, Viscera	ELISA, qRT-PCR, IFA	230	804	26	405	Cheng et al. (2025); Cui et al. (2013); Ding et al. (2014); Huang et al. (2019); Li et al. (2014); Niu et al. (2013); Wanshuang et al. (2024)
	Duck	Serum, Viscera	ELISA, qRT-PCR	12	61	9	61	Huang et al. (2019)
	Goat	Serum, Blood	ELISA, qRT-PCR	616	991	32	194	Cheng et al. (2025); Cui et al. (2013); Ding et al. (2014); Hu et al. (2019); Jiao et al. (2012); Li et al. (2014); Lu et al. (2025); Xing et al. (2016); Zhao et al. (2012)
	Geese	Serum	ELISA	2	120	–	–	Li et al. (2014)
	Hares	Serum, Viscera	ELISA, qRT-PCR	46	73	16	73	Huang et al. (2019)
	Hedgehog	Serum, Viscera	ELISA, RT-PCR, qRT-PCR	56	332	15	232	Hu et al. (2019); Huang et al. (2019); Jiao et al. (2012); Li et al. (2014, 2016); Sun et al. (2017); Zhao et al. (2022)
	Mink	Serum	ELISA	15	178	–	–	Wang et al. (2017)
	Pheasant	Serum, Viscera	ELISA, qRT-PCR	6	14	4	14	Huang et al. (2019)
	Pig	Serum, Viscera	ELISA, qRT-PCR	36	1369	29	902	Hu et al. (2019); Huang et al. (2019); Jiao et al. (2012); Li et al. (2014); Niu et al. (2013)
	Rock pigeon	Serum, Viscera	ELISA, qRT-PCR	8	23	5	23	Huang et al. (2019)
	Rodent	Serum, Viscera	ELISA, qRT-PCR, nested RT-PCR	67	2718	23	1577	Cheng et al. (2025); Gu et al. (2022); Hu et al. (2019); Huang et al. (2019); Li et al. (2014, 2016); Liu et al. (2014); Ni et al. (2015)
	Sheep	Serum, Viscera	ELISA, qRT-PCR	381	548	30	1232	Huang et al. (2019); Niu et al. (2013)
	Turtledoves	Serum, Viscera	ELISA, qRT-PCR	1	7	1	7	Huang et al. (2019)
	Wild boar	Serum	qRT-PCR	–	–	2	6	Kuan et al. (2023a)
	Yellow weasel	Serum, Viscera	ELISA, qRT-PCR	41	45	17	45	Huang et al. (2019)
Japan	Cat	Serum, Plasma, Oral/Rectal/Ocular swab, Blood	RT-PCR, qRT-PCR, ELISA, VNT, GLIPS	109	639	80	201	Ando et al. (2021); Kida et al. (2019); Matsuu et al. (2019, 2021, 2022); Osako et al. (2024); Sasaki et al. (2026); Yamanaka et al. (2020)
	Tsushima leopard cats	Serum	ELISA, VNT	1	6	–	–	Matsuu et al. (2022)
	Cattle	Serum	Indirect immunoperoxidase technique	11	510	–	–	Tabara et al. (2016)
	Cheetah	Plasma, Serum, Lymph node, Viscera	qRT-PCR	–	–	2	2	Matsuno et al. (2018)
	Deer	Serum	ELISA, VNT, GLIPS	552	916	1	229	Kimura et al. (2018); Matsuu et al. (2021); Tatemoto et al. (2022)
	Dog	Serum	ELISA, GLIPS, RT-PCR	18	245	1	114	Kimura et al. (2018); Matsuu et al. (2021, 2022)
	Japanese badgers	Blood, Viscera, Serum	qRT-PCR, ELISA	43	63	2	63	Kaneko et al. (2023)
	Raccoon dogs	Blood, Viscera, Serum	qRT-PCR, ELISA, VNT	12	53	–	–	Kaneko et al. (2023)
	Wild boar	Serum	ELISA, GLIPS, VNT, RT-PCR, FRNT, SRIP-neutralization	421	4063	9	207	Hayasaka et al. (2016); Kimura et al. (2018); Kirino et al. (2022); Matsuu et al. (2021, 2022); Tatemoto et al. (2022); Yazawa et al. (2025)
Korea	Cat	Serum	ELISA, RT-PCR, nested RT-PCR, IFA	11	203	35	564	Han et al. (2022); Hwang et al. (2017); Kang et al. (2020); Lee et al. (2017)
	Leopard cat	Spleen	RT-PCR	–	–	1	1	Byun et al. (2026a)
	Cattle	Serum	ELISA, nested RT-PCR	161	763	34	854	Chae et al. (2024)
	Chicken	Serum	ELISA, nested RT-PCR	83	296	7	312	Rim et al. (2023)
	Deer	Serum, Viscera	ELISA, IFA, RT-PCR, nested RT-PCR, VNT	45	236	12	450	Byun et al. (2024); Lee et al. (2020); Oh et al. (2016); Yu et al. (2019)
	Dog	Serum, Blood	ELISA, IFA, VNT, RT-PCR, qPCR, nested RT-PCR	86	542	21	1020	Byun et al. (2026b); Chung et al. (2020); Kang et al. (2019); Lee et al. (2017, 2018, 2020)
	Duck	Serum	ELISA, nested RT-PCR	81	246	3	249	Rim et al. (2023)
	Goat	Serum, Blood, Whole blood	ELISA, RT-PCR, nested RT-PCR	73	831	32	1694	Kang et al. (2018a); Ouh (2026); Yu et al. (2018)
	Horse	Serum	ELISA, VNT, RT-PCR	452	887	12	889	Han et al. (2023)
	Pig	Serum	nested RT-PCR	–	–	4	240	Kang et al. (2018b)
	Wild boar	Serum	ELISA, IFA, nested RT-PCR	119	848	50	867	Oh et al. (2016); Rim et al. (2021)
	Wild geese	Serum	ELISA, nested RT-PCR	8	26	5	45	Rim et al. (2023)
Thailand	Dog	Serum, Blood	VNT, RT-PCR	76	458	2	508	Ishijima et al. (2023)
	Rodent	Viscera	qRT-PCR	–	–	11	1019	Linsuwanon et al. (2024)
Taiwan	Sheep	Serum	qRT-PCR, nested RT-PCR	12	31	39	81	Kuan et al. (2023a), Lin et al. (2020)
	Goat	Serum	qRT-PCR	–	–	47	166	Kuan et al. (2023a), Lin et al. (2020)
	Cattle	Serum	qRT-PCR, nested RT-PCR	–	–	166	650	Kuan et al. (2023a)
	Wild boar	Serum, Viscera	qRT-PCR	–	–	2	6	Kuan et al. (2023a)
	Mammals (unspecified)	Serum, Viscera	qRT-PCR	–	–	4	257	Kuan et al. (2023a)
	Bird (unspecified)	Serum, Viscera	qRT-PCR	–	–	1	258	Kuan et al. (2023a)
	Dog	Serum, Plasma	RT-PCR	–	–	111	429	Kuan et al. (2023b)
	Cat	Serum, Plasma	RT-PCR	–	–	59	306	Kuan et al. (2023b)

ELISA, enzyme-linked immunosorbent assay; IFA, immunofluorescence assay; VNT, virus neutralization test; GLIPS, gaussia luciferase immunoprecipitation system; FRNT, focus reduction neutralization test; IPT, immunoperoxidase test; SRIP neutralization assay, single-round infectious particles neutralization assay; RT-PCR, reverse transcription polymerase chain reaction; qRT-PCR, quantitative reverse transcription polymerase chain reaction.

**Table 2.** Published reports of SFTSV isolation from animal hosts across East Asia

Host species	Specimen	Cell line	Virus isolate names	GenBank accession	Reference
Apodemus agrarius	Lung	Vero E6	rodent01 / Zhejiang / 2014, rodent02 / Zhejiang / 2014	KR698330–KR698331, KR698343–KR698344, KR698356–KR698357	Hu et al. (2019)
Domestic cat	Serum	Vero E6	Cat/Miyazaki/2018	LC462229–LC462237	Kida et al. (2019)
Erinaceus europaeus	Spleen	Vero E6, DH82 cells	JS2014-hedgehog-01 / 02	KR230768–KR230769, KR230788–KR230789, KR230808–KR230809	Li et al. (2016)
Cheetah	Plasma, spleen, lymph node, brain	Huh-7 cells, Vero E6 cells	SkrP/2017, ArtSp/2017	LC325234–LC325239	Matsuno et al. (2018)
Yellow weasel	Heart	Vero E6	-	MF574211–MF574213	Huang et al. (2019)
Domestic cat	Serum, mouse spleen homogenate	A129 mouse, Vero E6	NFe1, NFe3, NFe4, NFe8, NFe10, NFe11, NFe13, NFe37, NFe43, NFe45, NFe57, NFe66, NFe71, NFe76, NFe77, NFe79, NFe82, NFe88, NFe92, NFe93, NFe94, NFe97, NFe100, NFe101, NFe108, NFe111, NFe112, NFe114, NFe115	MN995224–MN995310	Matsuu et al. (2019)
Sheep, Cattle, Dog	Serum	Vero E6	SDLZSheep01/2011, SDLZCattle05/2011, SDPLDog01/2011	JQ693001–JQ693003	Niu et al. (2013)
Dog	Serum	Vero E6	Dog 22	KY968712, MH464251, MH464252	Kang et al. (2019)
Cat	Serum	Vero E6	P2021-03, P2021-09, P2021-31, P2022-03, P2022-06, P2022-08, P2022-10, P2022-13, P2022-17, P2022-29, P2022-31, P2023-06, P2023-12, P2023-13, P2023-18, P2023-24	PP273701–PP273716	Sasaki et al. (2026)
Cattle	Serum	Vero E6	-	OR987936–OR987938	Chae et al. (2024)
Korean water deer	Spleen	Vero E6	-	PP790964, PP790965, PP790966	Yu et al. (2019)
Dog	Serum	Vero E6	Dog 1, 12	OL773687, OK423754, OM179920–OM179923	Han et al. (2022)
Wild leopard cat	Spleen	Vero E6	KLC2025	PV816800, PV816801, PV816802	Byun et al. (2026a)
Cat	Serum	Vero E6	Cat 3, 4	OK423755, OL773688, OL773689, MZ342903, MZ352108, MZ363633	Lee et al. (2017)
